# Breast pain in the over 40s: impact on imaging

**DOI:** 10.1186/bcr3802

**Published:** 2015-11-05

**Authors:** Trupti Kulkarni, Andrew O'Connor

**Affiliations:** 1 Aintree University Hospital, Liverpool, UK

## Introduction

Current practice in our unit as agreed with the local Cancer Network Group is for women over 40 years presenting with breast pain and with a normal clinical examination to have a mammogram. NICE recommends no imaging in this group of patients. The aim was to measure workload impact from current practice, and assess diagnostic yield.

## Methods

Retrospective audit of imaging and biopsy in female patients over 40 years, presenting with breast pain, and who had normal clinical examination.

## Results

A total of 100 patients, aged 40−65, from 30 clinics over 3 months, 2014. Eighty normal mammograms. Seven of these had ultrasound for focal tenderness or probable glandular tissue, all of which were normal. Twenty abnormal mammograms: eight calcifications, six asymmetry, five discrete masses, one implant rupture. Total imaging workload: nine requests for previous imaging from elsewhere, eight further mammographic views, 11 ultrasounds, two stereo core biopsies (benign), one ultrasound-guided FNA followed by core biopsy (malignant). Yield: one cancer (25 mm grade 2 invasive ductal, negative sentinel lymph node).

## Conclusion

Workload is appreciably impacted by breast pain investigations. The final diagnosis was often delayed because of the wait for pathology results and previous imaging, increasing patient anxiety. The cancer detection rate number is too low for significance, but nevertheless compares favourably to screening. After discussion with clinicians it was decided to keep to our current practice as a means of opportunistic screening, particularly as our unit is in an area of poor screening uptake.

